# Comparison of Ultrasound Parameters and Clinical Parameters in Airway Assessment for Prediction of Difficult Laryngoscopy and Intubation: An Observational Study

**DOI:** 10.7759/cureus.41392

**Published:** 2023-07-05

**Authors:** Geetha Soundarya Udayakumar, Lakshmi Priya, Vidhya Narayanan

**Affiliations:** 1 Anesthesiology, Sree Balaji Medical College and Hospital, Chennai, IND

**Keywords:** anterior neck soft tissue thickness at thyrohyoid membrane, indian population, difficult laryngoscopy, preoperative ultrasonography, airway assessment

## Abstract

Background and objective

The primary responsibility of the anesthesiologist is to provide adequate oxygenation and ventilation to the patient by securing the airway. Prediction of Cormack-Lehane (CL) grading preoperatively helps patients’ airway management during anesthesia induction, particularly in difficult intubations. Our study aims to evaluate airway assessment modalities using ultrasound and conventional clinical screening methods for predicting difficult laryngoscopy and intubation.

Materials and methods

This prospective observational study was conducted on 100 patients aged between 18 and 70 years belonging to ASA classes I, II, and III scheduled for elective surgery requiring general anesthesia under endotracheal intubation was included in the study. Patients who needed rapid sequence induction and had a history of difficult intubation, obese patients with a body mass index (BMI) of more than 40, patients with notable swelling in the neck region (thyroid), pregnant patients, and patients with maxillofacial anomalies were excluded from the study. Clinical parameters such as body mass index, neck circumference, modified Mallampati grading, thyromental distance, and ultrasound parameters such as anterior neck soft tissue thickness at the level of the thyrohyoid membrane (ANS-TM) and anterior neck soft tissue thickness at the level of vocal cord (ANS-VC) were obtained preoperatively. After intubation, the CL grading was noted and categorized into two groups: easy (classes 1 and 2) and difficult (classes 3 and 4). Descriptive statistics included frequency and percentage for categorical variables and mean±standard deviation for continuous variables. The chi-square test was applied to find the relationship between easy and difficult laryngoscopy when compared with the outcome for categorical variables. A P value of less than 0.05 was considered significant throughout the study. The receiver operating characteristics curve (ROC curve) was used to determine the sensitivity and specificity to predict the outcomes.

Results

Ultrasound-guided measurements of ANS-TM and ANS-VC are independent predictors of difficult laryngoscopy compared with clinical screening tests. Of the two parameters, we found that ANS-TM has a better diagnostic value for predicting a difficult airway with an area under the ROC curve (AUC) of 91% compared with ANS-VC, which has an AUC of 84%. Of the clinical parameters, the modified Mallampati grading has an AUC of 81%, leading to better diagnostic value in the prediction of a difficult airway.

Conclusion

Our study demonstrated that ANS-TM and ANS-VC are independent predictors of a difficult airway. ANS-TM has a better correlation with CL grading. Clinical screening tests should be combined with ultrasound measurements to aid in the better prediction of difficult laryngoscopy.

## Introduction

Endotracheal intubation is routinely practiced to secure the airway during general anesthesia. Even well-experienced anesthesiologists can encounter challenges during unanticipated difficult intubation that can lead to failed intubation, which may increase morbidity and mortality. Routine preoperative airway assessment aids in the proper management of difficult airway conditions. Clinical screening tests used routinely in airway assessment lack adequate sensitivity and specificity for detecting difficult laryngoscopy. Cormack-Lehane (CL) grading view, obtained during direct laryngoscopy, is an invasive procedure and cannot be used for routine preoperative airway assessment [1].

Airway assessment using ultrasound during the preoperative period is a non-invasive diagnostic tool for measuring structures that may help predict a difficult airway [2]. Ultrasound can quantify almost all dimensions of airway structures, the same as a CT scan.

Many recent studies have highlighted the significance of various ultrasound airway measurements in predicting difficult intubation and showed a significant association between difficult laryngoscopy and different ultrasonic measurements [3,4]. Of these measurements, anterior neck soft tissue thickness at the level of the thyrohyoid membrane (ANS-TM) and anterior neck soft tissue thickness at the level of vocal cord (ANS-VC) had better predictive values, but with variation in their results [3,4]. In this regard, our primary objective was to evaluate the capability of ANS-TM and ANS-VC in predicting difficult laryngoscopy in Indian populations. The secondary objective was to compare ANS-TM, ANS-VC, and clinical airway screening tests-body mass index (BMI), neck circumference (NC), modified Mallampati (MMP) grade, and thyromental distance (TMD)-to predict difficult laryngoscopy and intubation.

## Materials and methods

This observational study was conducted in the Sree Balaji Medical College and Hospital anesthesia department from May 2020 to April 2021 after obtaining approval from the Institutional Ethics Committee (ref no: 02/SBMC/IHEC/2019/1291). Written informed consent was obtained from all the patients preoperatively.

Inclusion criteria

Patients aged 18-70 years with an American Society of Anesthesiologists (ASA) physical status of I, II, or III requiring endotracheal intubation under general anesthesia for elective procedures were included in the study.

Exclusion criteria

Patients who needed rapid sequence induction and had a history of difficult intubation, obese patients with a body mass index (BMI) of more than 40, patients with notable swelling in the neck region (thyroid), pregnant patients, and patients with maxillofacial anomalies were excluded from the study.

Sample size

Based on the study conducted by Wu et al. [4], the sample size was calculated based on the correlation between anterior neck soft tissue thickness at the level of thyrohyoid membrane and vocal cord, with 80% power, an alpha error of 5%, assuming a population correlation coefficient of 0.5%, and the total sample size calculated as 100 patients.

Data collection

The airway assessment of patients was performed in two phases: clinical parameters and ultrasound parameters. Basic demographics such as age, sex, height, weight, and BMI were noted. Clinical screening tests included MMP grading, NC, and TMD.

MMP grading was assessed with the patient sitting with the head in a neutral position, the mouth opened as much as possible, and the tongue protruding to the maximum.

The grading was based on the visible structures. Class I: soft palate, uvula, faucets, and anterior and posterior pillars were visible. Class II: soft palate, faucets, and uvula were visible. Class III: soft palate and base of the uvula alone were visible. Class IV: only the hard palate was visible.

The thyromental distance was measured with the patient made to lie flat in the supine position; the distance between the thyroid notch and the tip of the mentum was measured with the maximum possible extension of the neck. The neck circumference was calibrated with the help of a measuring tape around the neck at the level of the thyroid notch.

**Figure 1:**
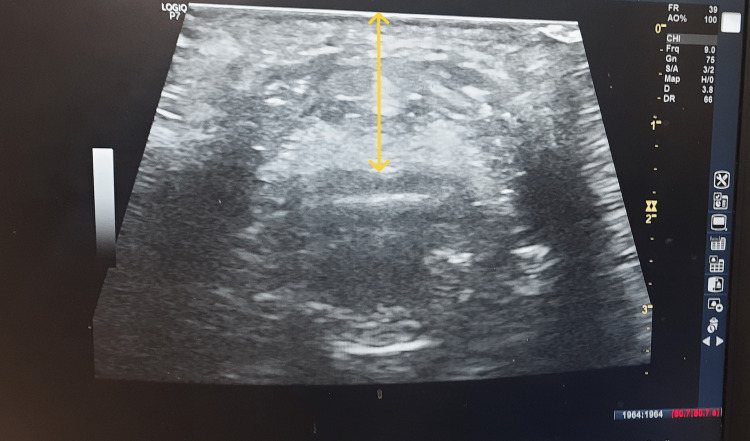
Ultrasound measurement of anterior neck soft tissue thickness at the level of thyrohyoid membrane.

Ultrasound airway assessment was performed using a General Electric-GE LOGIQ P7-sonography machine (GE Healthcare, USA) with 10-13 MHz frequency in the transverse plane, with the patient lying supine and keeping the head in a neutral position. ANS-TM was measured at the thyrohyoid membrane level (midway between the hyoid bone and thyroid cartilage). The distance from the skin surface to the middle axis of the highest part of the epiglottis through the thyrohyoid membrane was measured in centimeters (Figure 1).

**Figure 2:**
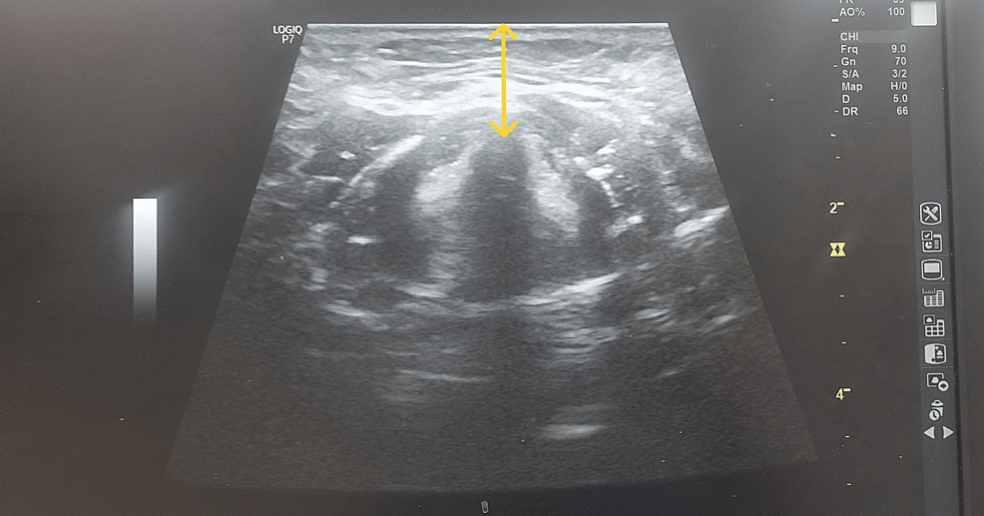
Ultrasound measurement of anterior neck soft tissue thickness at the level of vocal cord.

ANS‑VC was measured from the skin to the anterior commissure of true vocal cords with the linear probe placed transversely in the submandibular region; it was calculated by averaging the depth measured in the central axis of the neck in centimeters and 15 mm to the left and right (Figure 2).

The patient was then shifted to the operation theater; electrocardiogram, non-invasive blood pressure, pulse oximetry, and capnography monitors were connected. The patient was premedicated with intravenous injections of glycopyrrolate 0.005 mg/kg, midazolam 0.3 mg/kg, and fentanyl 2 mcg/kg, and then pre-oxygenated with 100% oxygen. The patient was induced with intravenous injections of propofol 2 mg/kg and atracurium 0.5 mg/kg and ventilated with oxygen and sevoflurane 2% for three minutes. A well-experienced anesthesiologist performed a direct laryngoscopy. The vocal cord was viewed without any external maneuver, and the anesthesiologist graded it as per CL grading. Intubation was graded easy (CL Grades 1 and 2) or difficult (CL Grades 3 and 4).

Cormack-Lehane's grading was based on visible structures. Grade 1: Entire vocal cords were visible. Grade 2: Partial view of vocal cords or arytenoids. Grade 3: Epiglottis alone was visible. Grade 4: Glottic structures were not visible at all.

Endotracheal tube position was confirmed with bilateral equal air entry and capnography. The anesthesiologist who intubated the patient was unaware of the preoperative ultrasound airway assessment. Anesthesia was maintained by nitrous oxide, oxygen at 50:50, sevoflurane 1%-2%, atracurium, and fentanyl as needed. After adequate recovery, the patient was reversed and extubated at the end of the surgery. The collected data were recorded and analyzed.

Statistical analysis

The data were tabulated using Microsoft Excel (Microsoft Corp., New York, USA) and analyzed using SPSS version 23 (IBM Corp., New York, USA) Descriptive statistics included frequency and percentage for categorical variables and mean±standard deviation for continuous variables. The chi-square test was applied to find the relationship between easy and difficult laryngoscopy when compared with the outcome for categorical variables. A P-value of less than 0.05 was considered significant throughout the study. The receiver operating characteristics curve (ROC curve) was used to determine the sensitivity and specificity to predict the outcomes. The AUC is a measure to assess the validity of the test. An AUC of one indicates a perfect diagnostic test.

## Results

A total of 100 patients (59 males and 41 females) were included in this study. The basic demographic characteristics are shown in Table 1. Using the CL grades assigned during direct laryngoscopy, the patients were divided into 82 patients (82%) in the easy group and 18 patients (18%) in the difficult group. Among the demographic details compared, body weight and BMI values were higher in the difficult laryngoscopy group. The mean body weight was 63.3±10.55 kg for the easy laryngoscopy group and 69.52±9.03 kg for the difficult laryngoscopy group (P < 0.05). The mean BMI value was 23.7±3.3 kg/m^2^ for the easy laryngoscopy group and 26.7±2.70 kg/m^2^ for the difficult laryngoscopy group (P < 0.05).

**Table 1:** Basic demographics of the patients with difficult and easy laryngoscopy. Data are presented as mean±SD or number of patients (%).

Parameters	Easy (n=82)	Difficult (n=18)	P-value
Age	42±12	44±13	0.529
Gender (M/F)	49(59.7%)/33(40.3%)	9(50%)/9(50%)	0.386
Height (cm)	162.9±8.2	165.3±7.3	0.254
Weight (kg)	63.3±10.55	69.52±9.03	0.0224
BMI (kg/m^2^)	23.7±3.3	26.7±2.7	0.0005

Clinical parameters such as NC, MMP, and TMD are shown in Table 2. The mean value of NC for the easy laryngoscopy group was 35±3 cm and 42±4 cm for the difficult laryngoscopy group (P < 0.001). A statistically significant difference was found between both groups for MMP grading (P < 0.001). The mean TMD for the easy laryngoscopy group was 6.49±0.82 cm, and 6.78±0.70 cm for the difficult laryngoscopy group (P = 0.48), which was not significant.

**Table 2:** Clinical parameters for predicting difficult laryngoscopy. Data are presented as mean±SD or number of patients (%). NC: neck circumference, MMP: modified Mallampati grade, TMD: thyromental distance.

Parameters	Easy (n=82)	Difficult (n=18)	P-value
NC	35±3	42±4	<0.0001
MMP			<0.0001
Class I	21(25.6%)	1(5.5%)	
Class II	44(53.7%)	8(44.4%)	
Class III	17(20.7%)	8(44.4%)	
Class IV	0(0%)	1(5.5%)	
TMD	6.49±0.82	6.78±0.70	0.48

The ultrasound parameters ANS-TM and ANS-VC are recorded in Table 3. The mean values of ANS-TM were 2.54±0.27 cm and 1.62±0.34 cm for easy and difficult laryngoscopy groups. The difference was significant, with a P value of <0.001. The mean value of ANS-VC was 0.94±0.18 cm for the easy laryngoscopy group and 1.42±0.28 cm for the difficult laryngoscopy group showing a significant P value of <0.001.

**Table 3:** Ultrasound parameters for predicting difficult laryngoscopy. Data are presented as mean±SD. ANS-TM: anterior neck soft tissue thickness at the level of thyrohyoid membrane; ANS-VC: anterior neck soft tissue thickness at the level of vocal cord.

Parameters	Easy (n=82)	Difficult (n=18)	P-value
ANS-TM	2.54±0.27	1.62±0.34	<0.0001
ANS-VC	0.94±0.18	1.42±0.28	<0.0001

The ROC analysis is depicted in Table 4. The ROC curve analysis illustrated that an ANS-TM of more than 2.03 cm is associated with difficult intubation, with an AUC of 0.91; ANS-VC of more than 1.12 cm is associated with difficult intubation, with the AUC being 0.84.

**Table 4:** The area under the ROC curves (AUC) for ANS-TM, ANS-VC, MMP, NC, and TMD. AUC±SE: area under the ROC curves±standard error; 95% CI: 95% confidence interval; ANS-TM: anterior neck soft tissue thickness at the level of thyrohyoid membrane; ANS-VC: anterior neck soft tissue thickness at the level of vocal cord; MMP: modified Mallampati grade; NC: neck circumference; TMD: thyromental distance.

Parameters	Optimal cut-off based on ROC	AUC±SE	95% CI	P
ANS-TM	2.03	0.91±0.04	0.86–0.97	<0.001
ANS-VC	1.12	0.84±0.01	0.80–0.91	<0.001
MMP	II	0.81±0.05	0.75–0.84	<0.001
NC	37	0.76±0.04	0.71–0.82	<0.001
TMD	6.54	0.51±0.02	0.45–0.54	0.48

We ascertained that MMP grade III is associated with difficult intubation. The optimal cut-off value for NC is 37 cm and for TMD is 6.54 cm for the prediction of difficult laryngoscopy. Based on the analysis of the ROC curve, the AUC for ANS-TM, ANS-VC, MMP, and NC was above 0.7 with a significant P value of 0.001, but the AUC for TMD was 0.51 and was not significant with a P value of 0.48.

## Discussion

Unanticipated difficult airways remain a challenge to anesthesiologists while securing an airway. Risk factors such as higher BMI, facial abnormalities, obstructive sleep apnea, limited cervical spine mobility, restricted mouth opening, MMP classes 3 or 4, a short TMD, and increased NC aid in predicting a difficult laryngoscopy, but none of them are 100% sensitive and specific. Ultrasound airway assessment is an emerging tool for predicting difficult airways. The tip of the laryngoscope blade lifts the tissues at the thyrohyoid membrane, and any increase in soft tissue thickness at this level increases CL grading. The MMP test is an indirect assessment of the thickness of the tongue. Difficult laryngoscopy is encountered in the case of an enlarged base of the tongue. The ultrasound measurements mainly reflect the thickness of the anterior neck soft tissue [5]. The combination of ultrasound and clinical parameters helps better predict unanticipated difficult laryngoscopy with increased sensitivity and specificity [6].

In this study, we compared the role of ultrasound assessment of airway parameters such as ANS-TM, ANS-VC, and clinical parameters (BMI, MMP, NC, and TMD) in predicting easy and difficult laryngoscopy in the Indian population. We found that the AUC for the ultrasound airway assessments was greater than that for the clinical airway assessment tools for determining difficult laryngoscopy. Among the ultrasound parameters, anterior neck soft tissue thickness at the level of thyrohyoid membrane had the highest AUC of 91% with a sensitivity of 97% and specificity of 79%; anterior neck soft tissue thickness at the level of the vocal cord had an AUC of 84% with sensitivity of 80% and specificity of 88%. Among the clinical parameters, MMP had the highest area of 81% under the ROC curve compared to other clinical parameters but lesser than that for ultrasound parameters suggesting that ultrasound airway assessments were better than the clinical screening tests in predicting difficult laryngoscopy.

We measured ANS-TM as part of our study; the values were 2.54 ± 0.27 cm and 1.62 ± 0.34 cm for easy and difficult laryngoscopy groups, respectively, with an optimal cut-off at 2.03 cm; the values above this were associated with difficult intubation and correlated significantly. Adhikari et al. [7] conducted a pilot study where they compared the association between ultrasound measurements (thickness of tongue and anterior neck soft tissue thickness at the levels of hyoid bone and thyrohyoid membrane) and clinical screening tests (MMP, TMD, and inter-incisor gap). They concluded that ultrasound measurement at the level of the thyrohyoid membrane with a cut-off at 2.8 cm was a good independent predictor of difficult laryngoscopy. The results were comparable to our study.

In their study, Saranya et al. [8] found an increase in soft tissue thickness at all three levels: the hyoid bone, the thyrohyoid membrane, and the suprasternal notch, which correlated with increased intubation difficulty. The thickness of anterior soft tissue from the skin to the epiglottis at thyrohyoid membrane level was 1.74 ± 0.26 cm for the easy laryngoscopy group and 2.38 ± 0.32 cm for the difficult laryngoscopy group, which is statistically significant (P < 0.001).

Bhagavan et al. [9] performed a pre-anesthetic ultrasound airway assessment. They concluded that distance from skin to epiglottis midway (DSEM)-thickness of anterior neck soft tissue in the transverse view at the level of the thyrohyoid membrane with a cut-off at 2.03 cm correlated positively with difficult laryngoscopy. As part of our study, we measured the patient’s thickness of ANS-VC. Measurements were higher in the difficult laryngoscopy group (1.42 ± 0.28 cm) than in the easy group (0.94 ± 0.18 cm), with a cut-off point of more than 1.12 associated with difficult intubation.

Ezri et al. [10] found in their study that increased neck tissue thickness at the vocal cord level was associated with difficult intubation, with a measurement of 2.7 cm for difficult and 1.8 cm for easy laryngoscopy; the difference was statistically significant (P < 0.001). Wu et al. [4] concluded that anterior neck thickness at the level of the anterior commissure was higher in the difficult laryngoscopy group, and they were significantly correlated. The results were similar to our study. As in our study, Yadav et al. [6] demonstrated a significant association between ANS-VC and difficult laryngoscopy.

In contrast to our study, Kesarwani et al. [11] conducted a study on 50 obese patients. They concluded that anterior neck soft tissue thickness at the level of the vocal cords was not significantly correlated with difficult intubation. Our study demonstrated that MMP grading of 3 and above was associated with difficult intubation with a P value of <0.001, with an AUC being 81%, which was lower when compared with ultrasound predictors. As MMP grading depends on the patient’s posture and cooperation, many studies have noted varying results [12]. A meta-analysis of 1,77,088 patients demonstrated that the modified Mallampati score (MMS) is inadequate as a stand-alone test for predicting difficult laryngoscopy. But it may be a combined clinical and ultrasound parameter to predict difficult tracheal intubation [13]. This conclusion was similar to our study.

Abdhelhady et al. [14] conducted a study similar to ours on 80 patients. They concluded that including ultrasound parameters such as MMP and TMD in routine clinical assessments helps to discriminate easy and difficult intubation compared to clinical parameters. In our study, NC of 37 cm and above were associated with difficult intubation with a P value of <0.001 and an AUC of 76%. Similar results were observed in the study conducted by Adhikari et al. [7], where mean NC of 50 ± 3.8 cm and 43.5 ± 2.2 cm were associated with difficult and easy laryngoscopy, respectively, with a P value of <0.001.

In our study, the mean TMDs for easy and difficult laryngoscopy were 6.49 ± 0.82 cm and 6.78 ± 0.70 cm, respectively, with the optimal cut-off at 6.54 cm. The AUC for TMD was 51%, with a P value of 0.48. It clearly shows that no significant association exists between TMD and difficult laryngoscopy. Tripathi and Pandey advocated that the normal value of TMD in adults is ≥6.5 cm [15]. Reddy et al. [16] observed that TMD did not help predict difficult intubation; this conclusion was similar to our study. However, systematic reviews [17,18] stated that commonly performed clinical screening tests such as the MMP score and TMD measurement have moderate accuracy and fail to discriminate between patients with difficult and easy airways consistently.

We have a few limitations to our study. Since we conducted this study on 100 Indian patients, the results cannot be generalized to other populations. The skill and experience of the anesthesiologists may influence CL grading and ultrasound measurements. While performing the ultrasound, the amount of pressure applied affects the values measured; this needs to be taken care of. We have excluded obese patients and parturients; these groups tend to have higher rates of difficult laryngoscopy, which needs to be studied in detail.

## Conclusions

We conclude that ultrasound-measured ANS-TM and ANS-VC help predict difficult airways better than clinical parameters. Anterior neck soft tissue thickness measured at the level of the thyrohyoid membrane was an independent predictor of difficult laryngoscopy supported by the highest AUC (91%) and sensitivity (97%). The commonly used clinical screening tests cannot be used as stand-alone tests to predict difficult airways and should be combined with ultrasound measurements to aid in the better prediction of difficult laryngoscopy.
